# Comparison of Surgical Outcomes for Finsterer and the Roux-en-Y Reconstruction after Distal Gastrectomy for Gastric Carcinoma

**DOI:** 10.1155/2021/5562776

**Published:** 2021-08-27

**Authors:** Pham Hoang Ha, Nguyen Xuan Hoa

**Affiliations:** ^1^Department of Digestive Surgery, Viet Duc University Hospital, 40 Trang Thi street, Hoan Kiem, Hanoi, Vietnam; ^2^Department of Surgery, University of Medicine and Pharmacy, Vietnam National University, Hanoi, 144 Dich Vong Hau, Cau Giay, Hanoi, Vietnam

## Abstract

**Objective:**

There have been surgical procedures to reconstruct the gastrointestinal continuity after distal gastrectomy. This study is aimed at comparing the surgical outcomes of reconstructing gastrointestinal continuity by the method of Finsterer and Roux-en-Y after distal gastrectomy due to cancer. *Materials and methods*. 86 patients, who underwent distal gastrectomy due to cancer, were divided into 2 groups for reconstructing gastrointestinal continuity from March 2014 to August 2018 at Viet Duc Hospital: group 1 (44 patients) by the Finsterer method and group 2 (42 patients) by the Roux-en-Y method.

**Results:**

The concentrations of bilirubin and amylase in gastric liquid after first flatus were 97.6 mmol/l and 20016 mmol/l for group 1 and 0.5 mmol/l and 152 mmol/l for group 2 (*p* = 0.01), respectively. The rate of reflux with clinical manifestations was 45.7% for group 1 and 9.4% for group 2 (*p* = 0.001). The average operation time was 155.7 ± 25.9 (90-200) minutes for group 1 and 170.3 ± 22.3 (120-215) minutes for group 2 (*p* = 0.007). The number of lymph nodes was 19.1 ± 4.8 (13-37) for group 1 and 20.3 ± 4.5 (12– 33) for group 2 (*p* = 0.243). There was 1 case of mesenteric bleeding in group 2 (2.4%). The dumping syndrome occurred in group 1 (20%) and group 2 (9.4%) (*p* = 0.31).

**Conclusion:**

The Finsterer and Roux-en-Y methods proved to be equally effective in their feasibility and safety. However, the Roux-en-Y method was better than the Finsterer method at limiting bile reflux and gastritis.

## 1. Introduction

There have been surgical procedures to reconstruct the gastrointestinal continuity after distal gastrectomy. A good reconstruction procedure should meet the requirements such as lowering postoperative complication rate, allowing patients to have a normal diet, improving quality of life [1]. Up to date, some common techniques have been recently reported, i.e., Billroth I, Billroth II, and Roux-en-Y. The advantage of Billroth I is to reconstruct the digestive tract physiologically but it has a higher rate of anastomosis leak. Technically speaking, it is difficult to carry out this technique because the remaining gastric remnant is required to be long enough (usually designated for cases of antrectomy, unfeasible to perform in the case of gastric cancer because resection margin must be ≥5 cm from the tumor) [2]. Therefore, the Billroth II and Roux-en-Y have been often selected after the distal gastrectomy because the patients can be operated on, regardless of the length of the remaining gastric remnant, and suffer a low rate of anastomosis leak. Although Billroth II (Finsterer) has been widely applied in Vietnam, it still has a disadvantage of duodenal reflux to the remaining gastric remnant and the esophagus, causing gastritis, oesophageal ulcers, and possibly recurrent cancer in this position [1]. The Roux-en-Y method was developed to limit bile reflux to the gastric anastomosis, but it may cause delayed gastric emptying symptoms after surgery (a.k.a. Roux syndrome) [3]. Makoto Ishikawa stated [1] that the Roux-en-Y method was more effective than the Billroth I method for prevention of gastric reflux and gastritis, but it could frequently cause gastrojejunal stasis leading to unnecessarily long postoperative hospital stays. According to a retrospective cohort study, i.e., all patients were followed up for 5 years [2], the Roux-en-Y method could be better than the Billroth I method in terms of minimizing the possibility of bile reflux into the gastric remnant and reflux esophagitis; although, there was no marked difference between these two methods in late complications (such as stomach ulcers, stomach bleeding, and stomach anastomosis narrowing) as well as nutritional status.

Due to the advancements in the treatment of gastric cancer, surgery may prolong the patient's life expectancy. Thus, it is necessary to evaluate the advantages and disadvantages of both Roux-en-Y and Finsterer anastomosis. Much data has been published on reconstructing the gastrointestinal continuity after distal gastrectomy, but little on comparing Finsterer and Roux-en-Y documented yet. This study is aimed at comparing the surgical outcomes of Finsterer and the Roux-en-Y reconstruction after distal gastrectomy for gastric carcinoma.

## 2. Subjects and Methods

- Subjects of the study

This retrospective comparative study selected 86 patients with 1/3 lower gastric carcinoma, which had got a distal gastrectomy in the Department of Digestive Surgery of Viet Duc Hospital from March 2014 to August 2018. Surgeries were performed by a group of specialists according to a unified surgical procedure. The studied patients were divided into two groups for the reconstruction of the digestive tract: group 1 by the Finsterer method and group 2 by the Roux-en-Y method.

(ii) Study method: this research was based on a retrospective comparative study
(iii) Surgical process

Step 1. Abdominal exploration: opening the abdomen by midline incision, i.e., a vertical incision which follows the linea alba to the navel, exploring the abdominal cavity, locating a tumor in the antropyloric zone (still indicated for partial gastrectomy), no metastasis detected, and organ invasion (radical surgery possibly feasible).

Step 2. Dissecting the gastrocolic omentum from the transverse colon (from right to left) to the inferior pole of the spleen; exposing and ligating the right gastroepiploic, right gastric, and left gastric vein; cutting and closing the upper part of the duodenum; performing a gastrectomy (with a distance of proximal resection margin >5 cm), removing the entire gastrocolic omentum; and performing an upper and lower gastric resection margin biopsy.

Step 3. Cleansing lymph nodes D2 (groups 1, 3, 4, 5, 6, 7, 8a, 9, 11p, 12a).

Step 4. Reconstructing the gastrointestinal tract by manual connection:

+ Finsterer: closing partly the gastric remnant and performing a side-to-side anastomosis of the first jejunal loop and the gastric remnant through the transverse mesocolon (the afferent loop placed at the lesser curvature).

+ Roux-en-Y: performing a Roux-en-Y anastomosis of the first jejunal loop and the gastric remnant through the transverse mesocolon (45-cm long Roux-en-Y loop used to avoid Roux syndrome), with the jejunum end placed at the greater curvature. The dissected jejunal loop is anastomosed end-to-side to the distal part of the jejunum 45 cm distal to the ligament of Treitz. A gastric catheter was inserted to get gastric juice for the postoperative test of bilirubin and amylase.

(iv) Research variables: comparing the surgical outcomes obtained by the Finsterer (Billroth II) and Roux-en-Y methods:

- Early results (during a 30-day postoperative period):

- Surgical duration and complications related to the anastomosis: mesenteric bleeding, anastomotic twisting (including the cases detected and remedied during operation), other organ damage, death in surgery, the number of dissected lymph nodes, length of hospital stay after surgery, postsurgical complications occurring within 30 days from surgery
- Testing data on amylase and bilirubin concentrations in the gastric juice on the first day after surgery and after the first flatus. Bilirubin and amylase concentrations were determined on the first day after surgery and after the first flatus

(b) Late results (after a 30-day postoperative period):

- Inflammation at the anastomosis: inflammatory changes at the anastomosis between the stomach and the proximal loop of the jejunum, evaluated by clinical symptoms of pain and stomach endoscopy
- Bile reflux: bile flows upwards from the duodenum into the stomach and esophagus through the gastric-jejunal anastomosis, evaluated by clinical symptoms of pain, endoscopic evidence of bile-stained fluid in the stomach, and bilirubin and amylase analysis in the stomach
- Dumping syndrome: when food, especially after a meal rich in sugar, moves too quickly from the stomach to the jejunum, evaluated by typical symptoms: abdominal pain, cramp, vomiting, diarrhea, and rapid or irregular heartbeat. The occurrence of this syndrome can be (i) 10-30 minutes after eating or (ii) 2-3 hours after eating

(c) Data analysis:

All statistical analyses were performed using SPSS ver.14.0. Categorical variables were analyzed using the *χ*^2^ test, and continuous variables were analyzed using the independent *t*-test. A *p* value of <0.05 was considered statistically significant.

## 3. Results

This study was conducted on a total of 86 patients (66.28% male and 33.72% female) selected and divided into 2 groups: group 1-radical gastrectomy, gastrointestinal reconstruction by the Finsterer method (44 patients); group 2-radical gastrectomy, gastrointestinal reconstruction by the Roux-en-Y method (42 patients). The average operating time was recorded to be 155.7 ± 25.9 (90-200) minutes for group 1 and 170.3 ± 22.3 (120-215) minutes for group 2 (*p* = 0.007).

Complication during surgery was only reported for one case of mesenteric hemorrhage in group 2 (2.4%). Postoperative hospital stay was shown to be 9.1 ± 1.8 (7-16 days) for group 1 and 8.9 ± 2 (6-15 days) for group 2 (*p* = 0.567).

Postoperative long-term follow-up results were available for 35/44 patients in the Finsterer group and 32/42 patients in the Roux-en-Y group. The postoperative follow-up time varied from 2 to 52 months (average: 22.7 ± 11.2 months). Gastroesophageal reflux symptoms were observed in 16 patients of group 1 (45.7%) and 3 patients of group 2 (9.4%) (*p* = 0.001). Tumoral recurrence at the anastomosis was seen in 2 patients of group 1 (4.5%) and 2 patients of group 2 (5%) (*p* < 0.05); distant metastasis for 5 patients of group 1 (11.4%) and 4 patients of group 2 (10%) (*p* > 0.05).

## 4. Discussion

Many surgical procedures have been reported for reconstructing gastrointestinal continuity after distal gastrectomy. The Finsterer method is routinely used as a simple anastomotic technique, but it still has disadvantages such as anastomotic biliary reflux and inflammation. The procedure of the uncut Roux-en-Y anastomosis was first proposed by Stiegman and Goff in 1988 [4]. Technically speaking, it is considered to be more difficult than the Billroth II method, but it can lower the rates of anastomotic biliary reflux and inflammation [5, 6].

It was shown that the rate of anastomotic biliary reflux (by an endoscopic evaluation) in the Roux-en-Y method (3.1%) was much lower than that in the Finsterer method (60%), *p* < 0.05 (Table 1). This biliary reflux reduction was also demonstrated by significantly lower concentrations of bilirubin and amylase in the stomach juice for the Roux-en-Y method on the day after surgery and at the time to first flatus (Table 2). This observation is believed to be due to the effect of the *Y*-loop [7, 8]. According to Prassana and colleagues, this anastomosis could lower the rate of gastroesophageal reflux from 26% to 2% [5]. It was also found that gastric juice pH value was <7 with the Roux-en-Y group and >7 with the Finsterer group, possibly indicating an amount of bile refluxed to the stomach through the anastomosis. It constitutes one of the risk factors for anastomosis cancer [7]. Our study also showed that the Finsterer group had a higher rate of inflammation than that of the Roux-en-Y group (i.e., 71.4% and 43.8% for the Finsterer group and Roux-en-Y group, respectively (*p* < 0.05), Table 1). It is in agreement with the meta-analysis carried out by Lirong He et al. reviewing 12 research studies (i.e., 4 randomized clinical trials and 8 nonrandomized clinical trials) revealing that the rate of anastomotic inflammation and biliary reflux of the Billroth II group was much higher than that of the Roux-en-Y group with *p* < 0.001 [8].

On the other hand, the dumping rate was not significantly different (*p* = 0.310) in both the Finsterer group (20%) and the Roux-en-Y group (9.4%). Late dumping syndrome was not detected in either group when the patients were distantly monitored (Table 3). It is somewhat different from other studies on Billroth II and Roux-en-Y reconstructions after distal gastrectomy clearly indicating a lower rate of dumping syndrome in the Roux-en-Y group compared to the Billroth-II group [9, 10]. In literature, it was shown that the Roux-en-Y method has a higher rate of gastric stagnation than that of the Finsterer method with symptoms of abdominal pain, vomiting, and nausea after eating (Roux syndrome). Gustavsson et al. found that up to 30% of patients in the Roux-en-Y group had symptoms of gastroparesis, especially with a Roux limb greater than 40 cm in length [8, 11]. Concerning this, in our study, no comparison was made for the two methods. According to the late outcomes, nonetheless, there was no marked difference in body weight change between these two groups of patients (Table 4).

Feasibility is particularly important in surgery, which is judged by operative time and blood loss. No significant difference in operative time was seen for the Roux-en-Y method (170.3 minutes) and Finsterer method (155.7 minutes) (Table 5), which is similar to the observation of Shuailong Yang in a meta-analysis [12] on these two groups *p* = 0.230. In contrast, other authors reported that operative time was longer in the Roux-en-Y group (244 minutes) than that in the Finsterer group (212 minutes) (*p* = 0.001) [13, 14]. For Roux-en-Y reconstruction, there are two anastomoses, i.e., a proximal gastrojejunalone and a distal jejunojejunalone. It means that the performance of *Y*-loop anastomosis may depend on factors such as surgeon experience, using or not using a stapler, anastomosis type (continuous or interrupted sutures). In this study, we used continuous sutures with a stapler so that it did not take much time to get a *Y*-loop anastomosis done additionally. Although blood loss was not mentioned in our study, Shuailong Yan [12] found that it should not be different between these two groups (*p* = 0.430).

The safety of a surgical method is demonstrated by mortality and complications that may occur during/after surgery. We only found 1 case of mesenteric bleeding during surgery that occurred in the Roux-en-Y group. Although the Roux-en-Y method was more technically difficult to perform [13, 15], there were no marked differences in postoperative complications such as anastomotic leak, wound infection, and postoperative abscesses between our two groups (Table 6). There was no postoperative mortality recorded in the two groups of patients.

## 5. Conclusion

Gastrointestinal reconstruction after partial gastrectomy for gastric carcinoma by the Billroth II and Roux-en-Y methods proved to be similar concerning surgical feasibility and safety. Nevertheless, the Roux-en-Y method is more effective at minimizing anastomotic biliary reflux and gastritis in the gastric remnant than the Billroth II method. Therefore, it is suggested that the Roux-en-Y method should be widely applied in surgical centers.

## Figures and Tables

**Table 1:** Gastric anastomosis status evaluated by endoscopy.

Group						
Indicator		Finsterer (*n* = 35)		Roux-en-Y (*n* = 32)		*p*
		*n*	%	*n*	%	
Anastomotic inflammation	Yes	25	71.4	14	43.8	0.027
	No	10	28.6	18	56,2	
Bile reflux	Yes	21	60	1	3.1	<0.01
	No	14	40	31	96.9	

**Table 2:** Concentration of bilirubin and amylase in gastric juice.

Group				
C (mmol)		Finsterer (*n* = 44)	Roux-en-Y (*n* = 42)	*p*
The first day after surgery	Bilirubin	26.25 (0.3-763)	2.14 (0-1270)	<0.01
	Amylase	23510 (0-377200)	1358 (1-126754)	<0.01
The day after the first flatus	Bilirubin	97.6 (0.3-936)	0.5 (0–548.3)	<0.01
	Amylase	20016 (0-152300)	152 (0-14310)	<0.01

**Table 3:** Dumping syndrome.

Group						
Syndrome		Finsterer (*n* = 35)		Roux-en-Y (*n* = 32)		*p*
		*n*	%	*n*	%	
Early dumping	Yes	7	20	3	9.4	0.310
	No	28	80	29	90.6	
Late dumping	Yes	0	0	0	0	
	No	35	100	32	100	

**Table 4:** Body weight change.

Group			
Body weight (kg) (mean ± SD)	Finsterer (*n* = 35)	Roux-en-Y (*n* = 32)	*p*
Before surgery	55.09 ± 6.87	53.10 ± 7.84	0.394
After surgery	54.34 ± 7.72	52.82 ± 7.07	
Change in body weight	0.75 ± 3.92	0.28 ± 3.47	

**Table 5:** Clinicopathologic characteristics of the two groups of patients.

	Finsterer (*n* = 44)	Roux en Y (*n* = 42)	*p*
Age (years)	55.2 ± 9.6 (35-72)	57.6 ± 11.6 (27-77)	>0.05
Male sex (%)	70.45	61.90	>0.05
BMI < 18 (%)	11.36	16.28	0.035
Time of operation (minutes)	155.7 ± 25.9 (90–200)	170.3 ± 22.3 (120–215)	0.007
Number of dissected lymphnode	19.1 ± 4.8 (13–37)	20.3 ± 4.5 (12–33)	0.243
Number of positive lymphnode	1.8 ± 2.5 (0–18)	2.6 ± 3.9 (0–10)	0.253
Tumor stage			0.631
T1a	2	2	
T1b	2	3	
T2	13	10	
T3	15	20	
T4a	12	7	
T4b	0	0	
Node stage			0.199
N0	22	20	
N1	9	8	
N2	12	7	
N3a	1	6	
N3b	0	1	
TNM stage			0.253
Ia	4	5	
Ib	12	4	
IIa	7	12	
IIb	5	4	
IIIa	7	10	
IIIb	9	6	
IIIc	0	1	
Tumor grade			0.22
Well-differentiated	6	4	
Moderately differentiated	19	26	
Poorly differentiated	19	12	

**Table 6:** Early outcomes after surgery.

Early results	Finsterer (*n* = 44)	Roux-en-Y (*n* = 42)	*p*
Time to first flatus (days)	3.6 ± 0.6	4.0 ± 0.6	0.5
Time for stomach catheterization (days)	4.9 ± 0.7	4.8 ± 0.8	0.391
Time to oral feeding (days)	5.7 ± 0.8	5.3 ± 1.0	0.054
Wound dehiscence (%)	1 (2.3%)	0	
Wound infection (%)	2 (4.5%)	5 (11.9%)	0.26
Postoperative intra-abdominal abscess	0	1 (2.4)	

## Data Availability

All data are available through the responsible corresponding author.

## References

[1] Ishikawa M., Kitayama J., Kaizaki S. (2005). Prospective randomized trial comparing Billroth I and Roux-en-Y procedures after distal gastrectomy for gastric carcinoma. *World Journal of Surgery*.

[2] Piessen G., Triboulet J. P., Mariette C. (2010). Reconstruction after gastrectomy: which technique is best?. *Journal of Visceral Surgery*.

[3] Hirao M., Osaka University Clinical Research Group for Gastroenterological Study, Takiguchi S. (2013). Comparison of Billroth I and Roux-en-Y reconstruction after distal gastrectomy for gastric cancer: one-year postoperative effects assessed by a multi-institutional RCT. *Annals of Surgical Oncology*.

[4] Van Stiegmann G., Goff J. S. (1988). An alternative to Roux-en-Y for treatment of bile reflux gastritis. *Surgery, Gynecology & Obstetrics*.

[5] Zong L., Chen P. (2011). Billroth I vs. Billroth II vs. Roux-en-Y following distal gastrectomy: a meta-analysis based on 15 studies. *Hepato-Gastroenterology*.

[6] Csendes A., Burgos A. M., Smok G., Burdiles P., Braghetto I., Díaz J. C. (2009). Latest results (12-21 years) of a prospective randomized study comparing Billroth II and Roux-en-Y anastomosis after a partial gastrectomy plus vagotomy in patients with duodenal ulcers. *Annals of Surgery*.

[7] Park J. Y., Kim Y. J. (2014). Uncut Roux-en-Y reconstruction after laparoscopic distal gastrectomy can be a favorable method in terms of gastritis, bile reflux, and gastric residue. *Journal of gastric cancer*.

[8] Lee M.-S., Ahn S.-H., Lee J.-H. (2012). What is the best reconstruction method after distal gastrectomy for gastric cancer?. *Surgical Endoscopy*.

[9] Wickremesinghe P. C., Dayrit P. Q., Manfredi O. L., Fazio R. A., Fagel V. L. (1983). Quantitative evaluation of bile diversion surgery utilizing ^99m^Tc HIDA scintigraphy. *Gastroenterology*.

[10] Lorusso D., Linsalata M., Pezzolla F. (2000). Duodenogastric reflux and gastric mucosal polyamines in the non-operated stomach and in the gastric remnant after Billroth II gastric resection. A role in gastric carcinogenesis?. *Anticancer Research*.

[11] Yang S., Chen F., Wang S. (2016). Meta-analysis and systemic review of different reconstruction methods for gastric carcinoma following distal gastrectomy. *EC Cancer 2.1*.

[12] He L., Zhao Y. (2019). Is Roux-en-Y or Billroth-II reconstruction the preferred choice for gastric cancer patients undergoing distal gastrectomy when Billroth I reconstruction is not applicable? A meta-analysis. *Medicine (Baltimore)*.

[13] In Choi C., Baek D. H., Lee S. H. (2016). Comparison between Billroth-II with Braun and Roux-en-Y reconstruction after laparoscopic distal gastrectomy. *Journal of Gastrointestinal Surgery: Official Journal of the Society for Surgery of the Alimentary Tract*.

[14] Tran T. B., Worhunsky D. J., Squires M. H. (2016). To Roux or not to Roux: a comparison between Roux-en-Y and Billroth II reconstruction following partial gastrectomy for gastric cancer. *Gastric Cancer*.

[15] Yang D., He L., Tong W.-H., Jia Z.-F., Su T.-R., Wang Q. (2017). Randomized controlled trial of uncut Roux-en-YvsBillroth II reconstruction after distal gastrectomy for gastric cancer: which technique is better for avoiding biliary reflux and gastritis?. *World Journal of Gastroenterology*.

